# GTADC: A Graph-Based Method for Inferring Cell Spatial Distribution in Cancer Tissues

**DOI:** 10.3390/biom14040436

**Published:** 2024-04-03

**Authors:** Tianjiao Zhang, Ziheng Zhang, Liangyu Li, Jixiang Ren, Zhenao Wu, Bo Gao, Guohua Wang

**Affiliations:** 1 College of Computer and Control Engineering, Northeast Forestry University, Harbin 150040, China; tianjiaozhang@nefu.edu.cn (T.Z.); zzhjs@nefu.edu.cn (Z.Z.); liangyuli@nefu.edu.cn (L.L.); 1241126773@nefu.edu.cn (J.R.); 2022112562@nefu.edu.cn (Z.W.); 2 Department of Radiology, The Second Affiliated Hospital of Harbin Medical University, Harbin 150040, China; 602192@hrbmu.edu.cn

**Keywords:** cancer, cell type identification, graph attention networks, single-cell RNA sequencing, spatial transcriptomics

## Abstract

The heterogeneity of tumors poses a challenge for understanding cell interactions and constructing complex ecosystems within cancer tissues. Current research strategies integrate spatial transcriptomics (ST) and single-cell sequencing (scRNA-seq) data to thoroughly analyze this intricate system. However, traditional deep learning methods using scRNA-seq data tend to filter differentially expressed genes through statistical methods. In the context of cancer tissues, where cancer cells exhibit significant differences in gene expression compared to normal cells, this heterogeneity renders traditional analysis methods incapable of accurately capturing differences between cell types. Therefore, we propose a graph-based deep learning method, GTADC, which utilizes Silhouette scores to precisely capture genes with significant expression differences within each cell type, enhancing the accuracy of gene selection. Compared to traditional methods, GTADC not only considers the expression similarity of genes within their respective clusters but also comprehensively leverages information from the overall clustering structure. The introduction of graph structure effectively captures spatial relationships and topological structures between the two types of data, enabling GTADC to more accurately and comprehensively resolve the spatial composition of different cell types within tissues. This refinement allows GTADC to intricately reconstruct the cellular spatial composition, offering a precise solution for inferring cell spatial composition. This method allows for early detection of potential cancer cell regions within tissues, assessing their quantity and spatial information in cell populations. We aim to achieve a preliminary estimation of cancer occurrence and development, contributing to a deeper understanding of early-stage cancer and providing potential support for early cancer diagnosis.

## 1. Introduction

Large-scale tumor genome projects have revealed extensive heterogeneity both between and within tumors [1]. The widespread application of single-cell RNA sequencing (scRNA-seq) technology has significantly enhanced our understanding of the heterogeneity features of tumor cells at the single-cell level [2]. However, scRNA-seq still has limitations, as the dissociation of tissues into single-cell suspensions results in the loss of spatial and morphological information, making it challenging to study the spatial structure of tumors [3,4,5]. Spatial transcriptomics (ST) technology overcomes these limitations, providing high-quality whole-genome transcriptomic data with complete two-dimensional positional information [6,7,8,9]. Combining ST and scRNA-seq helps overcome the limitations of each technique individually. Nonetheless, inherent limitations exist in spatial transcriptomic analysis, where, in most cases, each point or spot covers multiple cells. Even with high-resolution technology, a small fraction of several cells can be included in the same spatial barcode region. Moreover, tissues with high heterogeneity, such as cancer, consist of various cell types in each small region. There is a causal relationship between specific cell types in the tumor microenvironment (TME) and the state of cancer cells [10]. Therefore, identifying different cell types in spatial positions within cancer tissues is a crucial task, contributing to understanding the spatial context of pathological physiology.

To gain a deeper understanding of cell type distribution in spatial transcriptomics, current strategies predominantly involve integrating it with single-cell RNA sequencing (scRNA-seq). Mainstream approaches typically employ deconvolution techniques aiming to estimate the exact cell type proportions at each spatial position through regression models [11,12], deep learning models [13,14], or fitting probability distributions [15,16]. Feature selection is crucial for the performance of these deep learning models. However, current methods often rely on statistical approaches in feature extraction, selecting genes with the maximum differential expression among different cell types. Yet, in the process of extracting feature genes, cancer cell gene expression exhibits a more complex pattern. Specifically, the heterogeneous expression patterns of cancer cells significantly differ from normal cells, posing a challenge to accurately capture the tissue’s biological features. Understanding and considering this heterogeneity are crucial to ensure that the extracted feature genes accurately reflect the overall expression patterns of the tissue. Simultaneously, previous research has demonstrated that graph structures can effectively capture topological relationships and spatial correlations between spots. However, in cancer tissues, the spatial arrangement of cells presents complex heterogeneity [17,18]. Traditional simple graph methods fall short in adequately considering interactions and relative positions between spots. This heterogeneity accentuates the limitations of conventional graph methods, as they fail to capture the more intricate relationships between cells.

To overcome the limitations of traditional deconvolution methods in deciphering cell type distribution in cancer tissues, we introduce a novel approach named GTADC: a graph-based method for inferring cell spatial distribution in cancer tissues. This method employs a Graph Attention Network (GAT) model for deconvolution [19]. GTADC utilizes Silhouette scores, a metric based on the similarity and dissimilarity between cell types, to comprehensively capture differential expression patterns within each cell type [20,21]. This enables a deeper understanding and utilization of cell distribution and arrangement throughout the entire cancer tissue, thereby enhancing the accuracy and comprehensiveness of marker gene exploration. Our method incorporates the Seurat IntegrateData approach [22] to eliminate batch effects between generated pseudo-spatial transcriptomics (pseudo-ST) data and real ST data, significantly improving data consistency and comparability [23].

Subsequently, we employ a random projection forest to construct a weighted adjacency matrix [24], accurately characterizing the topological relationships between pseudo-ST and real ST. Through a complex graph structure representation, our method adapts better to the heterogeneity in gene expression within cancer tissues. Importing these features and the weighted adjacency matrix into the GAT model achieves accurate inference of cell type composition at each position in spatial transcriptomics. Validated on simulated spatial transcriptomics data, GTADC demonstrates high accuracy and sensitivity in predicting cell type proportions. This innovative approach not only enables the early detection of potential cancer cell regions within tissues but also accurately determines the content and positional information of these cells within cell populations. Through this technology, we anticipate providing a preliminary estimation of cancer occurrence and development, offering robust potential support for early cancer diagnosis, and providing a comprehensive and insightful perspective for a deeper understanding of the cancer microenvironment.

## 2. Materials and Methods

### 2.1. Datasets

GTADC takes as input spatial transcriptomics (ST) data, gene expression data from single-cell RNA sequencing (scRNA-seq), and cell type metadata for each cell. Due to the difficulty in obtaining reliable standards from real ST datasets for method validation, we chose to generate a benchmark testing ST dataset using two scRNA-seq datasets from human colorectal cancer (GSE132465 and GSE144735) [25]. In this validation process, scRNA-seq data from colorectal cancer patients in Korea and Belgium were used to simulate the ST dataset fully randomly to assess the performance of GTADC. To simulate the ST dataset, we randomly selected mixtures of 2–8 cells from scRNA-seq, adding their gene expression values to represent the gene expression for each spot in the synthetic ST data. This synthetic ST data not only mimics the characteristics of real ST data but also provides a reliable benchmark for evaluating GTADC’s performance in identifying different cell type proportions within each synthetic spot. This process helps ensure that GTADC can accurately and reliably infer cell spatial distribution in the face of real cancer tissues.

To comprehensively evaluate GTADC’s performance and reliability in inferring cell spatial distribution in real cancer environments, we tested it on three different types of cancer tissues. First, we selected human squamous cell carcinoma, using ST data and scRNA-seq data from the study GSE144240 [26]. Second, we examined human hepatocellular carcinoma, where ST data came from the study “Comprehensive analysis of spatial architecture in primary liver cancer” [27], and scRNA-seq data originated from GSE149614 [28]. Due to the non-correspondence of the stages between ST and scRNA-seq data for this cancer, resulting in significant differences in the number of each cell type, especially in cancer cell types, we adopted a combination of multiple samples in this cancer. We performed random sampling for each cell type with a fixed quantity, using the sampled results as reference scRNA-seq data for the experiment. Finally, we studied human breast cancer, with scRNA-seq data from GSE176078 and ST data obtained from the public database https://zenodo.org/records/4739739 (accessed on 5 June 2021), patient ID CID4535 [29]. The diverse set of cancer samples helps validate GTADC’s generality and robustness in different cancer environments.

### 2.2. Implementation of GTADC

**Figure 1:**
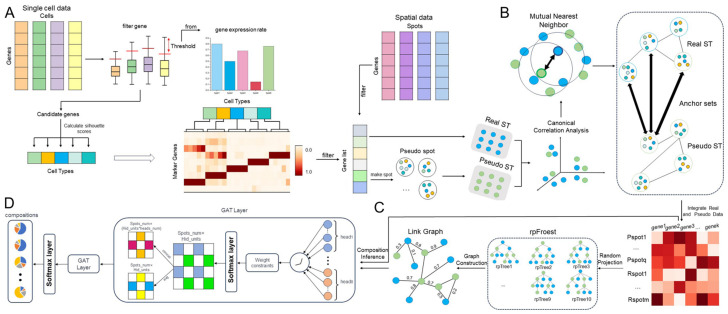
The framework diagram depicts the utilization of GTADC in deconvolving spatial transcriptomic data of cancer tissue. (**A**) Extract the most representative genes from the gene expression matrix of cancer tissues as features. (**B**) Integrate the generated pseudo-ST and real ST data. (**C**) Obtain the topological relationships between spots based on the random projection forest. (**D**) Utilize the processed gene expression matrix and adjacency matrix as inputs to train the model and determine the proportions of cell types within spots.

Feature selection plays a crucial role in GTADC. In contrast to other deep learning methods that tend to focus on genes with significant differential expression in each cell type or select a subset of genes with the most significant differential expression overall, GTADC employs a more flexible and adaptive feature gene selection method tailored to the heterogeneity of cancer tissues. Traditional methods, such as statistical rank tests in tools like Seurat or scnapy [30,31], often struggle to accurately filter genes with significant meaning in cancer tissues due to the complex and diverse nature of gene expression abnormalities. To address this challenge, GTADC introduces an innovative feature selection strategy that better adapts to the heterogeneity of cancer tissues. Additionally, as the graph structure is a crucial data structure for representing topological relationships, a more nuanced modeling approach is required for cancer tissues. Traditional k-nearest neighbors (knn) methods may struggle to meet the complexity of data relationships we encounter in cancer tissues. Therefore, GTADC employs a novel graph construction method and an improved Graph Attention Network (GAT) model to accurately predict the cell type proportions for each spot. This integrated strategy of feature selection and graph modeling enhances GTADC’s robustness and accuracy when dealing with cancer tissue data. The overall workflow of GTADC is depicted in the figure below (Figure 1).

#### 2.2.1. Feature Genes Selection

In classical scRNA-seq datasets, there are typically over 20,000 genes. To reduce computational complexity, the expression rates of all genes in each cell type are first computed. As a preprocessing step before filtering, genes with expression levels below 20% across all cell types are removed. Subsequently, normalization is performed to mitigate experimental biases for effective comparisons [32]. The gene expression in scRNA-seq data is represented as $Q\in R^{n\times m}$, where *n* is the number of cells, and *m* is the number of genes. The matrix *Q* is considered a combination of *k* distinct cell types, each treated as an independent cluster. For enhanced computational efficiency, gene filtering is applied to each cluster. In the gene filtering stage, the expression rate matrix *O* and the mean expression value matrix *P* are computed on the *Q* matrix. Utilizing the calculated expression rate matrix *O*, genes with expression rates above a threshold are filtered for each of the *k* cell types, as expressed by the following formula:

(1) $$\mathrm{threshold}=0.5\times \left(2\times Q_{3}+1.5\times IQR\right)$$

Here, *Q*_3_ represents the third quartile, and *IQR* stands for the interquartile range (the difference between the third and first quartiles, i.e., between the 75th and 25th percentiles).

Next, filtering is performed on the expression rate matrix *O* and the average expression value matrix *P* to obtain $O_{1}\in R^{k\times s}$ and $P_{1}\in R^{k\times s}$, where *s* indicates the number of genes with expression rates higher than the threshold. Simultaneously, filtering is applied to *O*_1_ and *P*_1_ using the top-ranking genes in the average expression matrix *P*_1_ to obtain $O_{2}\in R^{k\times l}$ and $P_{2}\in R^{k\times l}$.

For each gene *g*, calculate a dispersion measure to obtain candidate genes. Subsequently, rank the genes based on their dispersion values, selecting the top *t* genes with the highest dispersion values as candidate marker genes for the *k* cell types. The formula is as follows:

(2) $$\mathrm{disp}=\frac{(a-b)\times c^{e}}{d^{*}}$$

In this context, *a* represents the average expression level of gene *g* in cell type *k* (derived from matrix *P*_2_), *b* signifies the maximum average expression level of gene *g* in other cell types (from matrix *P*_2_), *c* denotes the expression rate of gene *g* in cell type *k* (derived from matrix *Q*_2_), *d* represents the average expression rate of gene *g* in other cell types (from matrix *Q*_2_), and *e* is a constant regulating the importance of the expression rate of gene *g*.

In cancer tissues, the heterogeneity of cell types and states is pronounced, and single-cell RNA sequencing (scRNA-seq) data often reflects this heterogeneity. In such cases, traditional statistical methods may fail due to substantial variations in gene expression, as these methods assume that the data are identically distributed. In contrast, the Silhouette coefficient, as a non-parametric method based on similarity and distance, exhibits greater robustness to the heterogeneity of cancer tissues. Therefore, the use of the Silhouette coefficient in GTADC serves as a metric for evaluating clustering quality. It provides a measure of clustering effectiveness ranging from −1 to 1 [33]. This coefficient is based on the similarity of each data point to other points within the same cluster and the dissimilarity to the nearest points in the neighboring cluster. For each cell type, the top *t* genes with the highest Silhouette coefficients are selected, forming the final set of feature genes.

#### 2.2.2. Pseudo-ST and True ST Data Integration

In previous approaches, multiple cells were typically combined to simulate the composition of real ST (spatial transcriptomics) data. In this step, the gene expression values of multiple cells were often summed to form the gene expression of a single spot, creating pseudo-ST data. These pseudo-ST data were generated by mixing single-cell RNA sequencing (scRNA-seq) data from the same tissue, aiming to emulate spatial transcriptomics (ST) data. The purpose of generating pseudo-ST data is to provide a theoretically controllable dataset for evaluating and optimizing the performance of deconvolution methods.

We followed the process of generating a benchmark test dataset using scRNA-seq to create pseudo-ST data as a reference for model training. To enhance the consistency and comparability between the generated pseudo-ST data and true ST data, we integrated pseudo-ST and true ST data. Initially, cells underwent preprocessing and standardization, typically involving embedding cells from each dataset into a low-dimensional space. Common methods, such as Principal Component Analysis (PCA), were employed to reduce computational complexity and extract key cell features. Subsequently, we computed similarity scores between each pair of spots (sample points). Typically, cosine similarity or other similarity measures were used to assess the degree of expression pattern similarity between spots. Between the two datasets, we selected spot pairs with high similarity scores as anchor points. These anchor point pairs represented spots with similar expression patterns between different datasets. Then, we associated the anchor points by maximizing the similarity scores to determine which spots corresponded between different datasets. This process aimed to establish connections between datasets, preserving the information of anchor points. Leveraging the information of anchor points, we aligned spots from different datasets, mapping them to a common integrated space. This involved mapping features from each dataset to the integrated space and consolidating them into an overall expression matrix. Finally, by integrating different datasets, we formed a unified expression matrix. This integration method effectively fused cell expression information between different datasets, providing a robust foundation for subsequent comprehensive analyses.

#### 2.2.3. Graph Construction

First, create an index array to track the original order of feature data, preserving data order during the construction of the random projection tree [34]. Next, build a random projection tree based on the integrated feature matrix and return the root node of the tree [35,36]. Obtain a list of indices for all leaf nodes. For each leaf node, combine the points within that node pairwise, generating an edge list. This list encompasses all edges between points within the same leaf node. Set the weight of all edges in the edge list to 1 and convert it into an adjacency matrix. Subsequently, add the adjacency matrix of each random projection tree to the overall adjacency matrix to integrate the results of all trees. By averaging the results of all trees, the weighted adjacency matrix obtained represents the final result. In comparison to traditional k-nearest neighbors (knn) methods, this approach demonstrates superior accuracy and performance in constructing the graph structure of cancer tissue. Not only does it enhance precision, but it also exhibits robust processing capabilities when dealing with highly heterogeneous cancer tissue data, enabling a more detailed and accurate analysis of cancer tissue data.

#### 2.2.4. Model Building

Compared to traditional graph convolutional networks (GCN), Graph Attention Network (GAT) [37] exhibits significant advantages in addressing the specificity of cancer tissue. GCN employs fixed weights when aggregating node information, assigning equal importance to the contribution of each node to its neighboring nodes. This rigid weight assignment may lack flexibility when dealing with the highly heterogeneous and complex relationships among nodes in cancer tissue. GAT cleverly addresses this issue by introducing a graph attention mechanism. By calculating adaptive attention weights, the model dynamically learns the importance and correlation between nodes. This mechanism takes into account the diversity of relationships between different nodes, providing the network with more flexible expressive capabilities. Specifically, GAT leverages the relationships between the graph structure and node features to adaptively learn the degree of association between each node and its neighboring nodes by computing attention weights. This allows the model to more accurately capture the complex biological characteristics of cancer tissue, as interactions between different cells may have varying importance in different contexts. A notable advantage is that the GAT model incorporates multiple graph attention heads. Each head can learn different attention weight distributions between nodes, comprehensively capturing contextual information for nodes. This multi-head mechanism enables the model to simultaneously consider and integrate information from multiple relationships, enhancing its ability to model complex relationships.

In the model, for each attention layer, given *N* node features, $h|=\left\{{\vec{h}}_{1},{\vec{h}}_{2},\ldots ,{\vec{h}}_{N}\right\},{\vec{h}}_{i}\in R^{F}$, where each node feature has a dimension of *F*, the calculation of attention coefficients can be represented as:

(3) $$e_{ij}=\left({\vec{a}}^{T}\left[W{\vec{h}}_{i}∥W{\vec{h}}_{j}\right]\right),$$

where $e_{ij}$ represents the attention coefficient of node *i* with respect to node *j*, and ${\vec{a}}^{T}$ and *W* are both shared learnable parameters.

Subsequently, after calculating the attention coefficients, the previously computed adjacency matrix weights are incorporated into the model, emphasizing the topological relationships of the graph. The formula is represented as follows:

(4) V=E⊙A

Here, *V* represents all attention coefficients taking into account the weights of the adjacency matrix, *E* represents the attention coefficients between all nodes, and *A* represents the overall weighted adjacency matrix. 

By parallelly computing multiple attention heads, the model comprehensively captures information in the input sequence. The outputs of all attention heads at each layer are concatenated as input for the next layer, or an average operation is performed at the final layer to obtain the model’s output result:

(5) $$h_{i}^{\prime }={∥}_{k=1}^{K}\sigma \left(\underset{v_{j}\in N\left(v_{i}\right)}{\sum }\alpha_{ij}^{\left(k\right)}W^{\left(k\right)}h_{j}\right)$$

Here, $h_{i}^{\prime }$ represents the new feature of node *i* after incorporating neighborhood information, ∥ denotes vector concatenation or averaging operations, and σ represents the activation function.

The cross-entropy is employed as the loss function to evaluate the pseudo-ST data, and the softmax activation function is utilized in the output layer to learn the cell type proportions. Upon completion of training, the model provides estimates of cell type proportions for all spots in the test data, mimicking the real ST data.

## 3. Results

### 3.1. Evaluate Algorithm Performance in Comparison with State-of-the-Art Methods

In this study, we employed a cell mixing approach to generate a synthetic dataset as our benchmark data, based on the human colorectal cancer dataset used for benchmarking in Section 2.1. In this benchmark dataset, the proportions of cell types are known, enabling a comprehensive evaluation of the method’s performance in inferring cell spatial distribution.

We conducted a comparison of GTADC with other published tools for cell type deconvolution, including CellDART [13], STRIDE [16], DSTG [14], and RCTD [15]. Specifically, we compared GTADC with GTAD [38], which replaces the feature gene selection stage with a statistical test (implemented in the scanpy package). When running each of the previously published methods, all parameters were set to their default values as specified in their documentation. This approach allowed us to comprehensively evaluate the performance of GTADC in the feature gene selection stage and understand its advantages relative to other methods. For performance comparison, we utilized the Jensen–Shannon distance (JSD) as a benchmark metric [39]. JSD is a distance metric used to measure the similarity between two probability distributions, ranging from zero to one. A JSD value of zero indicates complete similarity between two distributions, while a value of one indicates complete dissimilarity with no overlap. Therefore, a smaller JSD value indicates higher similarity between the estimated cell type compositions, reflecting higher accuracy. This comprehensive performance comparison framework provides insights into the performance of GTADC relative to other tools.

**Figure 2:**
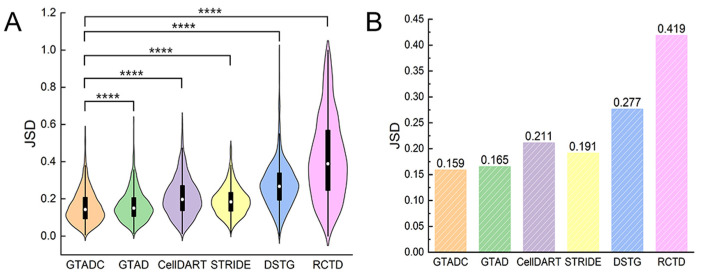
The performance of GTADC on benchmark datasets. (**A**) Violin plots comparing the performance of various methods on the simulated dataset of human colorectal cancer. In order to ensure that the comparative figures appear aesthetically pleasing and fully displayed, we have set the lower limit of the y-axis for JSD (Jensen–Shannon divergence) to extend below zero. The JSD values themselves range from 0 to 1. (**B**) Bar chart depicting the average JSD of simulated spatial data generated from human colorectal cancer scRNA-seq dataset. Wilcoxon signed-rank test was conducted on JSD values of GTADC against other methods. Statistical significance (**** *p*-value < 10^−4^) is indicated at the top of violin plots in (**A**).

Based on the experimental results, we generated violin plots and bar charts using the Jensen–Shannon distance (JSD) as the metric. In the bar chart, the average JSD of GTADC is significantly lower than other published methods, especially compared to GTAD, indicating superior performance (Figure 2B). The violin plot provides a more intuitive visualization, showing that GTADC has more data points in the regions with lower JSD, implying smaller differences between estimated cell type proportions and actual proportions at these points (Figure 2A). To ensure the reliability of the results, we conducted Wilcoxon rank-sum tests. The Wilcoxon rank-sum test, a non-parametric statistical method, is used to compare the distributions of two independent samples for significant differences [40]. In comparison to the t-test, the Wilcoxon rank-sum test makes no assumptions about the distribution of data, making it suitable for non-normally distributed data. The results demonstrate that GTADC significantly outperforms other methods in terms of JSD, with all comparisons yielding *p*-values less than 0.0001, providing strong statistical support for our conclusions.

In summary, our study indicates that GTADC exhibits outstanding performance and stability in deciphering spatial transcriptomics data, providing a reliable tool for accurately interpreting the cell type composition in cancer tissues. This has significant implications for advancing our understanding of the cancer microenvironment and the development of precision medicine.

### 3.2. Decomposition of Spatial Cell Distribution with GTADC in cSCC

Squamous cell carcinoma (cSCC) is a prevalent type of skin cancer, and studying it contributes to a deeper understanding of the pathogenic mechanisms of skin tumors. Analyzing cSCC data allows us to identify expression characteristics of different cell types, understand immune cell infiltration, and explore interactions within the tumor microenvironment. As a widely occurring tumor type, studying cSCC provides essential clues for early detection of the disease and improvement of treatment methods [41]. The application of GTADC to cSCC data helps unveil the cellular composition and transcriptomic features of this tumor, laying the foundation for a comprehensive understanding of its development.

**Figure 3:**
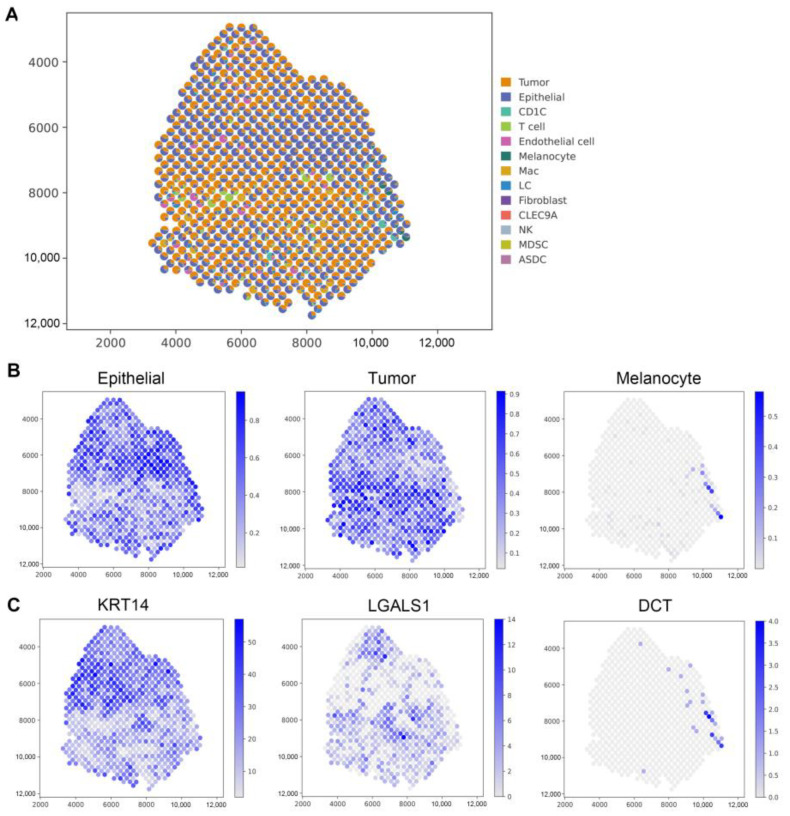
Application of GTADC on cSCC. (**A**) Spatial distribution of all predicted cell types by GTADC. Each point represents an ST spot, with colors indicating different cell types. (**B**) Spatial distribution of selected cell types, including epithelial cell, tumor cell, and melanocyte. (**C**) Spatial mapping of marker genes for each cell type in (**B**). Color mapping represents the maximum and minimum values of corresponding cell scores.

We selected data from patient 2 for spatial deconvolution of the ST data, successfully reconstructing the structure of cSCC (Figure 3A). A pie chart intuitively displays the proportions of heterogeneous cells identified at each local spot, confirming not only the accuracy of predictions but also highlighting the high predictive accuracy and sensitivity of the GTADC method. Furthermore, the GTADC method successfully predicts and spatially maps the distribution of important cell types.

Moreover, the GTADC method provides detailed information on the composition of each cell type, enhancing our understanding of heterogeneity. The research results indicate a correlation between the enrichment of each cell type in the region and its determined proportion (Figure 3B). Additionally, as the original study did not provide explicit proportion results, we mapped the gene expression distribution of marker genes corresponding to cell types in that spatial domain for validation [42], confirming the accuracy of the GTADC experimental results. For instance, epithelial cells are enriched in the upper-middle part of the slice, consistent with the high expression of its marker gene *KRT14* in that region; tumor cells mainly concentrate at the top and middle-lower parts of the slice, with sparse clustering in other areas, aligning with the expression trend of the *LGASL1* gene in that region; melanocytes, with fewer in number, are minimally enriched at the far right of the slice, where its marker gene *DCT* shows high expression, undetectable or at very low levels in other spatial points (Figure 3C).

GTADC’s spatial deconvolution in cSCC enables precise analysis, uncovering cell type distribution and interactions. This innovative approach offers insights into tumor dynamics, aiding targeted therapies. It sets a precedent for comprehensive cancer microenvironment studies.

### 3.3. Application of GTADC on Hepatocellular Carcinoma

Hepatocellular carcinoma (HCC) is the most common malignant tumor of the liver globally and typically accompanies the development of chronic liver diseases. In-depth analysis of HCC data allows a comprehensive study of the molecular mechanisms of liver cancer and exploration of the expression characteristics of tumor cells in liver tissue. Given the high incidence and invasiveness of HCC, molecular research is crucial for identifying effective treatment methods [43]. The application of GTADC to HCC data provides a profound understanding of the transcriptomic heterogeneity of liver cancer, establishing a solid foundation for the development of personalized treatment and diagnostic strategies.

**Figure 4:**
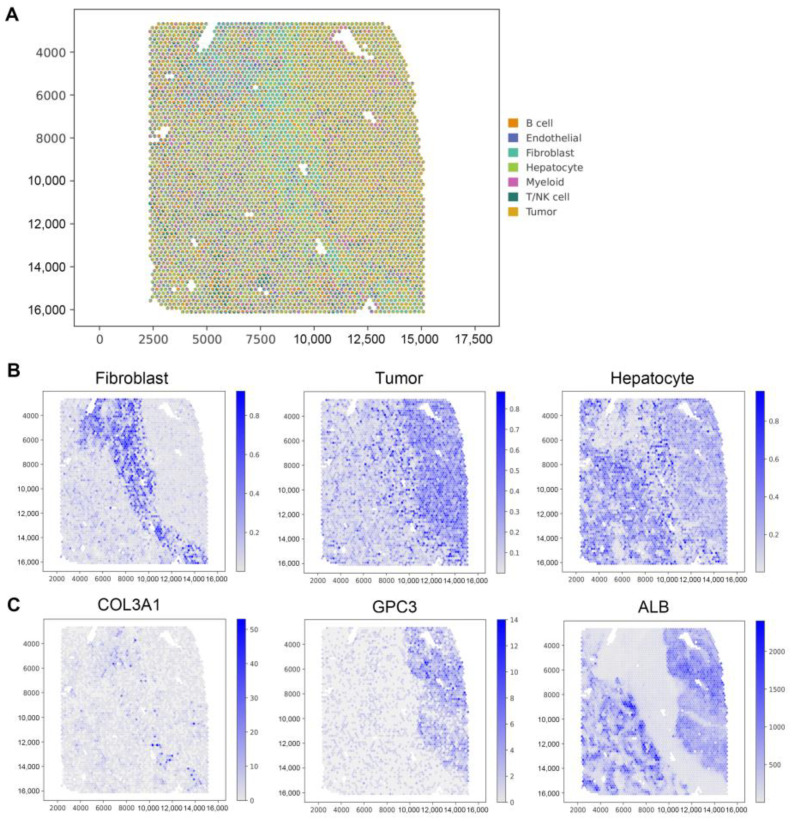
Analysis of the HCC SRT data. (**A**) Visualization of deconvolution results. Spatial scatter pie charts depict the predicted cell type composition by GTADC, with each point representing a spot in the SRT data. (**B**) Visualization of the abundance of selected cell types at each spatial position. (**C**) Display of marker gene expression levels for the cell types corresponding to (**B**).

Due to the distinct origins of scRNA-seq and ST data from different studies, covering various stages of cancer tissue and varying cancer cell quantities in different stages, the heterogeneity of cancer tissue and the differing cancer cell numbers in different stages can significantly impact the semi-supervised GTADC method. To address this challenge, we merged sequencing results from multiple samples in scRNA-seq data and performed fixed-sample-size random sampling for each cell type, using the sampling results as a reference dataset. The results of the GTADC method were utilized to map the reference cell types to the spatial positions in the images (Figure 4A). This strategy enables a more accurate revelation of the distribution and spatial relationships of different cell types in human primary liver cancer. This method provides a powerful tool for a deeper understanding of the cellular features of HCC.

According to the original study, we observed significant distribution differences among the “transition zone” and its adjacent “normal zone” and “tumor zone”. The deconvolution results of GTADC for this cancer are consistent with the literature description (Figure 4B). In the “transition zone”, fibroblasts were mapped to the center of the small sample, clearly distinguishing the cancer and normal areas, aligning with the distribution of its marker gene *COL3A1*. Tumor cells were significantly enriched on the right side of the sample, consistent with the location of the high expression of the marker gene *GPC3*, in line with the original study’s description of the “tumor zone”. Hepatocytes were widely distributed in the tissue, scattered in both the “normal zone” and “tumor zone”, with a higher quantity in the “normal zone” than in the “tumor zone”, corresponding to the distribution range of the marker gene *ALB* (Figure 4C).

In Figure 4, while the fibroblast results exhibit clear signals that align with the biological experiments of the original dataset, the classification boundaries for the other two cell types lack clarity, indicating less distinct boundaries between them. This finding suggests that our method may encounter challenges in accurately delineating boundaries between these cell types based solely on gene expression data. This is primarily because single cell gene expression data may not fully capture the complex cellular phenotype differences. Cells in different physiological states and microenvironments may exhibit similar gene expression patterns, leading to blurred classification boundaries. Therefore, for more accurate delineation of cell type boundaries, future research may need to integrate multiple data sources, such as protein expression data and cellular morphological features, to comprehensively consider the multidimensional information of cells.

GTADC excelled in deconvoluting HCC spatial transcriptomics, unveiling diverse cell types’ distribution and relationships. This deep analysis reveals HCC’s unique features, holding potential for understanding its pathogenesis, finding new targets, and personalized treatments.

### 3.4. GTADC Characterizes the Spatial Heterogeneity of Tumor Cells in Human Breast Cancer

**Figure 5:**
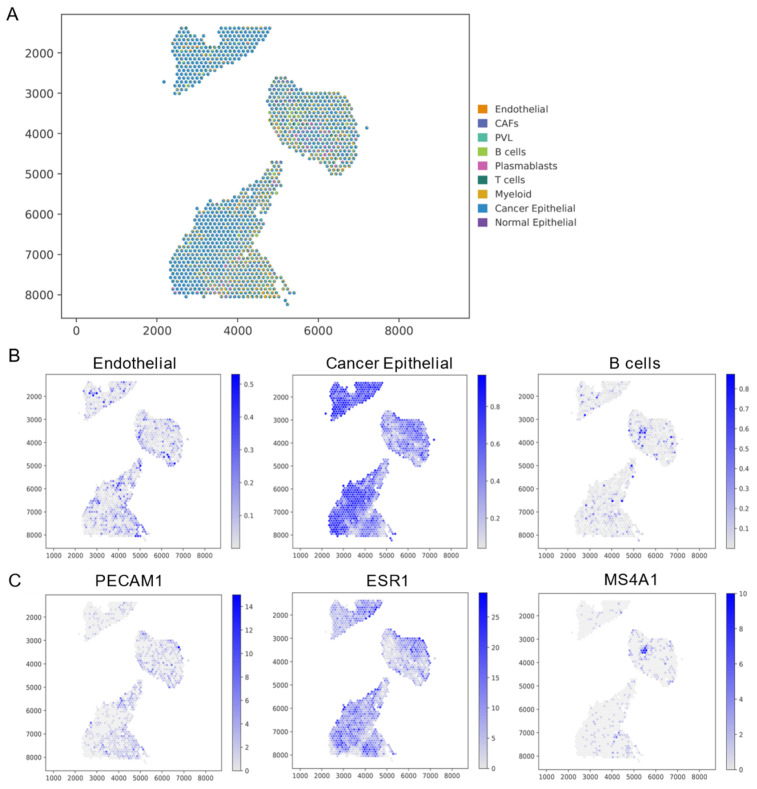
Analysis of human breast cancer tissue cells using GTADC. (**A**) Spatial distribution of all predicted cell types by GTADC. Each point represents a captured pixel, with colors indicating different cell types. (**B**) Spatial distribution of endothelial, cancer epithelial, and B cells. Color mapping represents the maximum and minimum cell scores. (**C**) Display of marker gene expression levels for the cell types corresponding to (**B**).

Breast cancer, one of the most prevalent cancers among women, may also occur in men. In-depth analysis of breast cancer data allows a better understanding of tumor heterogeneity, molecular subtypes, and the impact of the tumor microenvironment on cancer development. One key goal of breast cancer research is to identify the molecular characteristics of different subtypes, providing a solid scientific foundation for personalized treatment [44]. The application of GTADC in breast cancer data is significant as it helps reveal the transcriptomic differences among various cell types in breast tissue, providing a foundational support for more precise diagnostic and treatment strategies (Figure 5A). This research direction holds promise for improving treatment outcomes and survival rates for breast cancer patients.

By examining the spatial distribution of cell types inferred by GTADC and comparing it with the original study, which focused on the distribution of marker genes specific to each cell type, we gained insights into the accuracy of GTADC’s predictions (Figure 5B). In breast cancer tissue, we observed that endothelial cells were located in the peripheral regions, consistent with the spatial expression pattern of the marker gene *PECAM1*. The distribution of cancer epithelial aligned with the source of the ST data, being highly enriched in the upper tissue, predominantly present in the central tissue’s edge regions, and exhibiting a distinct layered structure in the lower tissue, closely resembling the expression pattern of the marker gene *ESR1*. Finally, B cells were sparsely present in the tissue, clustering in a few spots in the central tissue, consistent with the expression pattern of the marker gene *MS4A1* (Figure 5C). Moreover, the expression levels of these cell type-specific marker genes in other spots were lower or undetectable. These observations further validate the reliability of GTADC in accurately revealing the distribution of cell types in breast cancer tissue.

GTADC’s precise analysis in breast cancer deepens cell distribution understanding, showcasing exceptional efficacy. This method supports cancer research, laying a solid foundation for future strategies, providing a unique view into breast cancer’s microcosm.

## 4. Discussion

In current biological research, cancer, as a compelling focus of study, benefits significantly from the combined analysis of multi-omics technologies, providing a powerful tool to delve into the essence of this disease [45]. The complexity and heterogeneity of cancer make it challenging to reveal the complete picture relying solely on data from a single omics approach. The integration of multi-omics methods addresses this limitation, enabling a more comprehensive understanding of the various changes in cancer occurrence, progression, and treatment processes [46,47,48]. By integrating information from single-cell genomics and spatial transcriptomics, we can gain a more accurate insight into the interactions between different cell types in tumor tissue. This integrated analysis not only helps uncover the intricate complexity of cell signaling pathways but also facilitates in-depth exploration of the distribution and mutual influences of cells in the tumor microenvironment. This holistic multi-omics approach plays a crucial role in cancer research, offering opportunities for a more comprehensive understanding of tumor biology. Furthermore, such integrated analyses aid in identifying potential therapeutic targets, providing more precise directions for personalized treatment [49]. Therefore, multi-omics integration studies in cancer not only expand our theoretical knowledge of cancer biology but also establish a solid foundation for future treatment strategies and drug development.

In this study, we introduced an innovative method, GTADC, to gain a deeper understanding of cancer, a complex disease. We implemented a unique feature selection mechanism designed to more accurately identify genes with a greater inclination for classification. This innovative feature selection method not only contributes to improving the performance of the classification model but also flexibly adapts to the heterogeneity of cancer tissue, allowing us to finely explore potential biological information. Simultaneously, by integrating two types of data, we significantly enhanced data consistency and comparability, making it more accurate for application in spatial transcriptomics. Additionally, we employed a more complex graph construction method, namely, a random projection forest, to comprehensively describe the complex relationships between data. The heterogeneity of cancer tissue is not only reflected in the differences in gene expression but also in the complexity of interactions between cells. By introducing graph structure, we better capture and represent the unique relationships between different parts of cancer tissue, providing the model with richer information. The application of a random projection forest enables us to more comprehensively and accurately reveal the complex biological features within cancer tissue, offering a new perspective for a deeper understanding of cancer.

## 5. Conclusions

In summary, GTADC exhibits unique advantages in addressing the spatial composition of cells in cancer tissue, demonstrating high accuracy and sensitivity in predicting cell type proportions. It serves as a reliable tool for in-depth exploration of spatial cell composition issues. The distinctive features of GTADC provide a new perspective, unlocking fresh possibilities in cancer research. It stands out as a powerful instrument, contributing to a deeper understanding of cancer biology in the scientific community.

## Data Availability

All the data used in this study are publicly available datasets. The datasets analyzed during the current study are available in the GEO repository GSE132465, GSE144735, GSE144240, GSE176078, GSE149614. The datasets analyzed during the current study are available in the Zenodo repository, study [29], https://zenodo.org/records/4739739 (accessed on 5 June 2021). The datasets analyzed during the current study are available in the repository, study [27], http://lifeome.net/supp/livercancer-st/data.htm (accessed on 17 December 2021). The Python and R source codes for GTADC have been uploaded to https://github.com/zzhjs/GTADC (accessed on 30 November 2023).
